# Treatment of Ozœna

**Published:** 1876-04

**Authors:** 


Treatment of Ozœna.—Dr. A. d'Azambuja first draws attention to the fact that the term ozœna only indicates a symptom, namely the fœtid odor of the air expired through the nose, a symptom that may occur in varions forms of coryza, and which he has satisfied himself may be observed in chronic ozœna without any ulceration. In most instances ozœna results from some constitutional affection, as from scrofula or syphilis, and as a sequela of various forms of infectious diseases; occasionally it is a consequence of eczema of the mucous membrane, injuries, etc., in persons who are otherwise in sound health. In regard to its treatment he chiefly recommends the use of the nasal douche and the direct cauterization of the ulcer, if any ulcer be present. Necrosed portions of bone must be removed. It is only in very extreme cases that he can admit the applicability of Roughe's severe operative proceeding (reflection of the whole upper lip after division of the septum nasi through the mucous membrane of the nose), Rouge having lost one case in nine operations from meningitis.—*Centralblatt f. d. Chirurgie*, No. 38, 1875.—*London Practitioner*, January, 1876.

